# Supplementary figures and images for: Genome-scale CRISPR screening at high sensitivity with an empirically designed sgRNA library
Source: BMC Biol. 2020 Nov 23;18:174. doi: 10.1186/s12915-020-00905-1 (PMC7686728; doi:10.1186/s12915-020-00905-1)

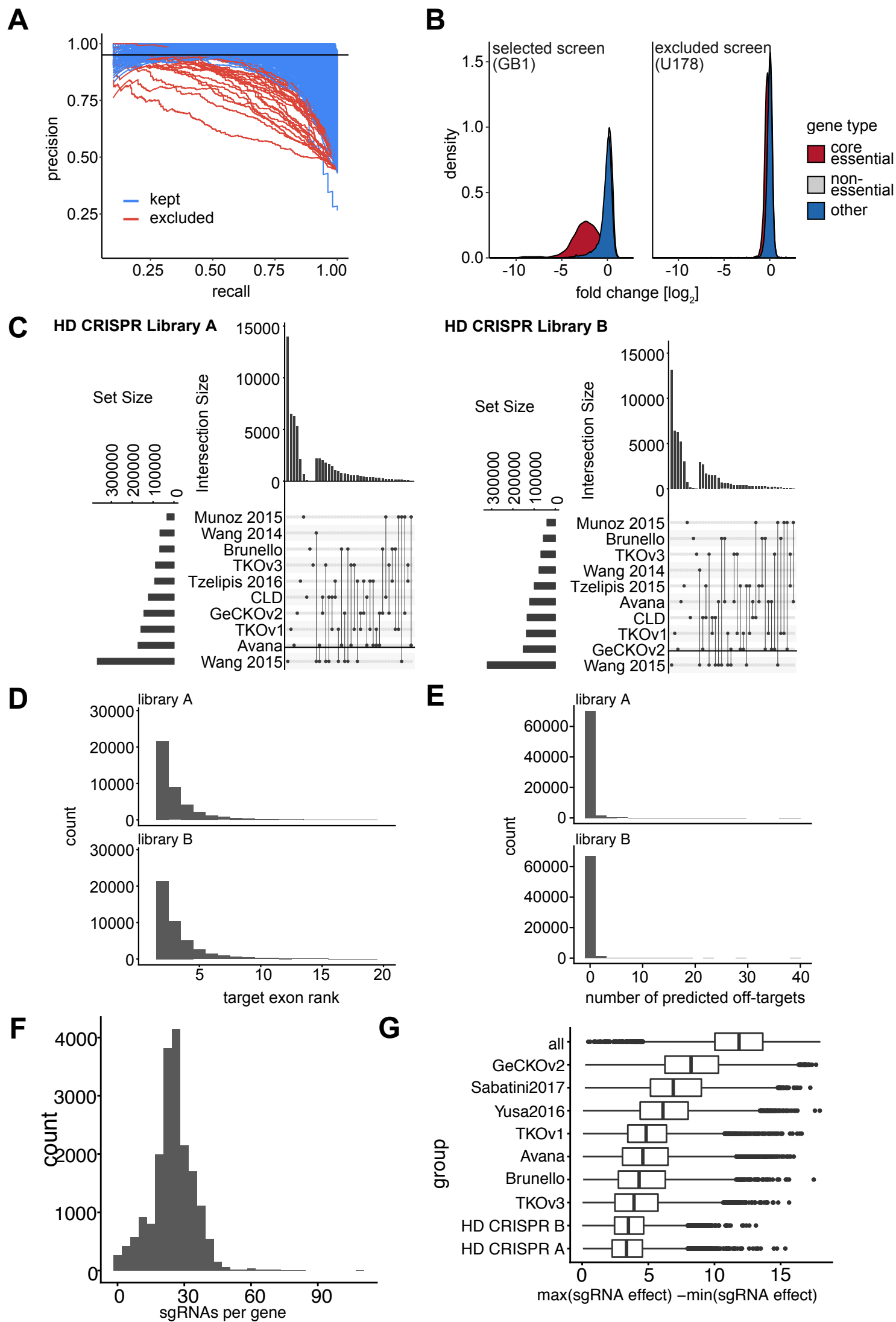

**Figure S1**

Supplement: Supplementary file 1 — Additional file 1: Figure S1. HD CRISPR library composition. (A) Precision recall curves for differentiating reference core and nonessential genes based on BAGEL Bayes Factors determined for published fitness screens in GenomeCRISPR. Blue curves indicate screens with an area under the curve (AUC) greater than 0.9. Red screens with an AUC of less than 0.9 were excluded for HD CRISPR sgRNA design. (B) Log2 fold change distributions of core essential (red) and nonessential (blue) reference genes for a high quality (left) and a low quality (right) example screen. (C) Library composition of the HD CRISPR sub-libraries A and B. Horizontal bars on the left indicate the number of designs used from different previously published libraries. The panel on the bottom right shows combinations of libraries, that include designs selected for the HD CRISPR library. The bars above this panel quantify the number of selected sgRNAs for each of these combinations. (D) Distribution of exon ranks targeted by the sgRNAs in the HD CRISPR library. (E) Distribution of the predicted off-target counts (see Materials & Methods) for sgRNAs in the HD CRISPR library. (F) Number of sgRNAs, which remained per gene after pre-filtering and were considered for library design. (G) Phenotypic deviation of published sgRNA phenotypes targeting the same gene. For each gene the difference between the GenomeCRISPR effect scores of the sgRNAs with the smallest and the largest effect scores was calculated. This process was repeated for each library using only those sgRNAs included in that library. Guides selected for the HD CRISPR libraries A and B show a narrow phenotypic deviation in published screens from which they were selected. [file 12915_2020_905_MOESM1_ESM.pdf]

**A**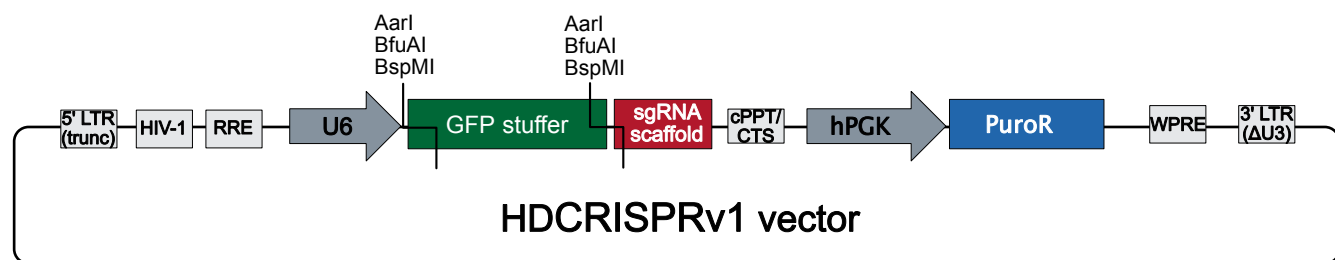**B**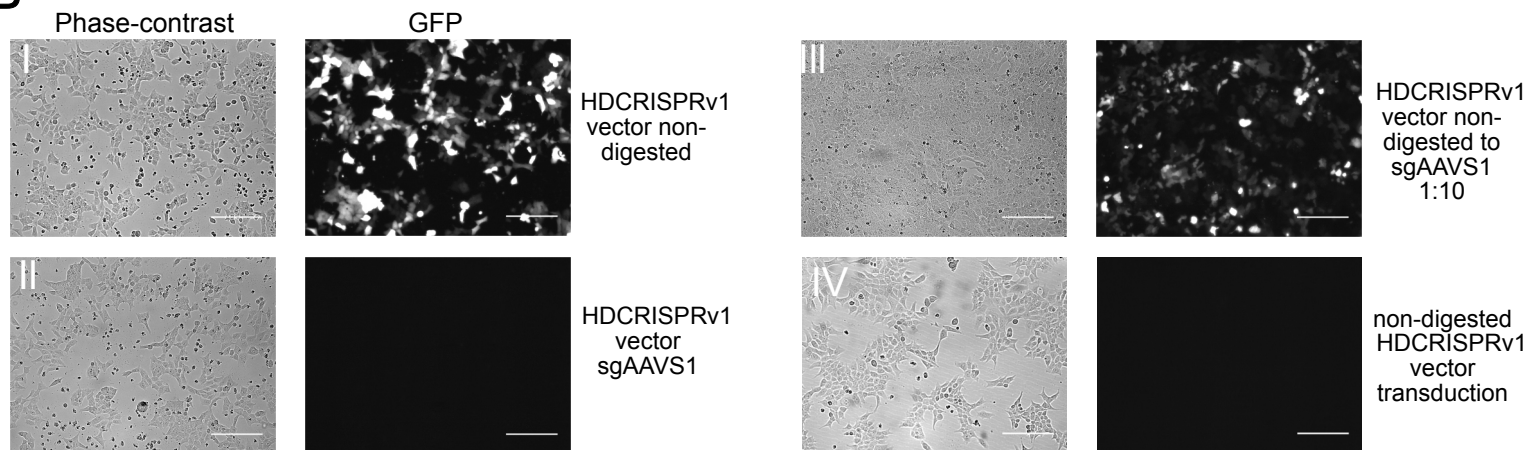**C**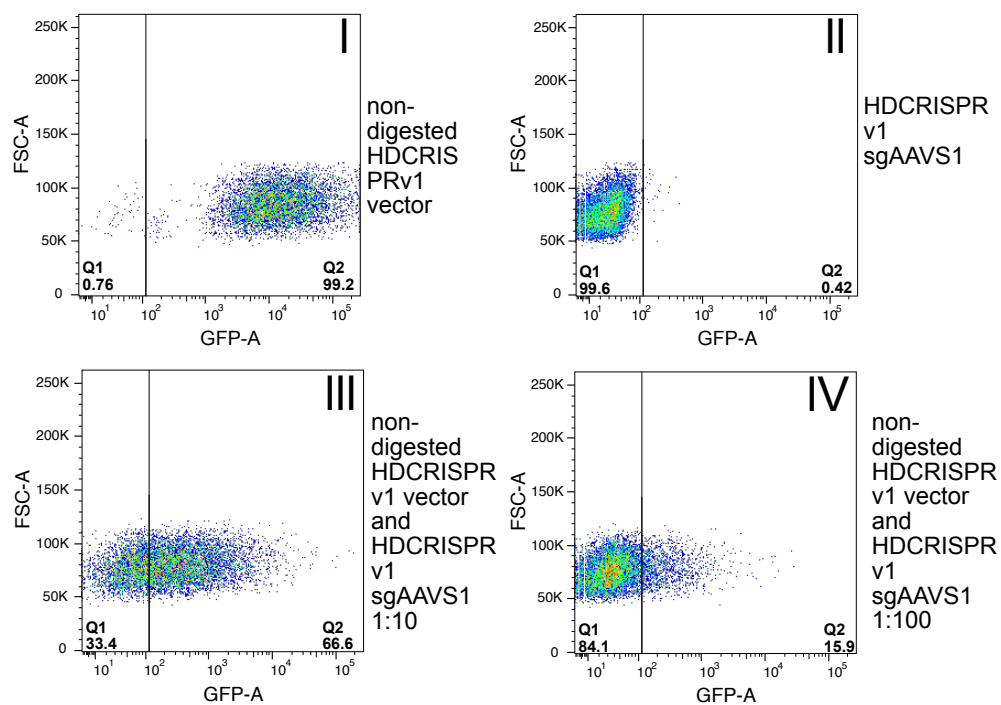**D**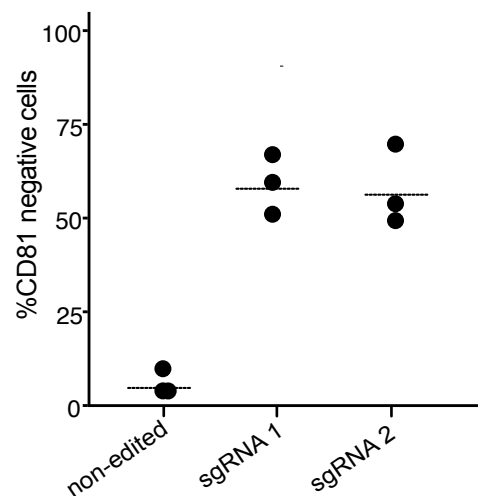**Figure S2**

Supplement: Supplementary file 5 — Additional file 5: Figure S2. Features and performance of the HDCRISPRv1 vector. (A) Composition of the lentiviral HD CRISPR sgRNA expression vector. (B) sgRNA cloning efficiency can be addressed upon transfection of the HDCRISPRv1 vector, since residual GFP stuffer in non-digested vector backbone leads to GFP expression (B.l) (n = 2). Complete removal as achieved when cloning single sgRNAs abolishes GFP expression (B.ll) (n = 2), while remaining stuffer in 10% of the plasmid pool still leads to a substantial amount of GFP positive cells (B.lll) (n = 2). Transduction of the non-digested vector still containing the GFP-stuffer does not result in GFP-expressing cells (B.IV) (n = 1). Scale bar = 100 μM (C) FACS analysis of GFP expression upon transfection of the non-digested HDCRISPRv1 vector (l) (n = 2) or the HDCRISPRv1 vector expressing an sgRNA (ll) (n = 3). A mixture of GFP positive and negative cells can be observed upon transfection of a mixture of stuffer and sgRNA-containing vector (lll and IV) (n = 3 for III and n = 2 for IV). (D) Editing efficiency was furthermore assessed upon transduction of HAP1 Cas9 cells with the HDCRISPRv1 vector expressing sgRNAs targeting the surface proteins CD81, followed by FACS staining of residual CD81 protein to address knockout efficiency. Antibody staining of the non-edited cell line was used as a control. Lines represent the mean of three independent experiments for each condition. [file 12915_2020_905_MOESM5_ESM.pdf]

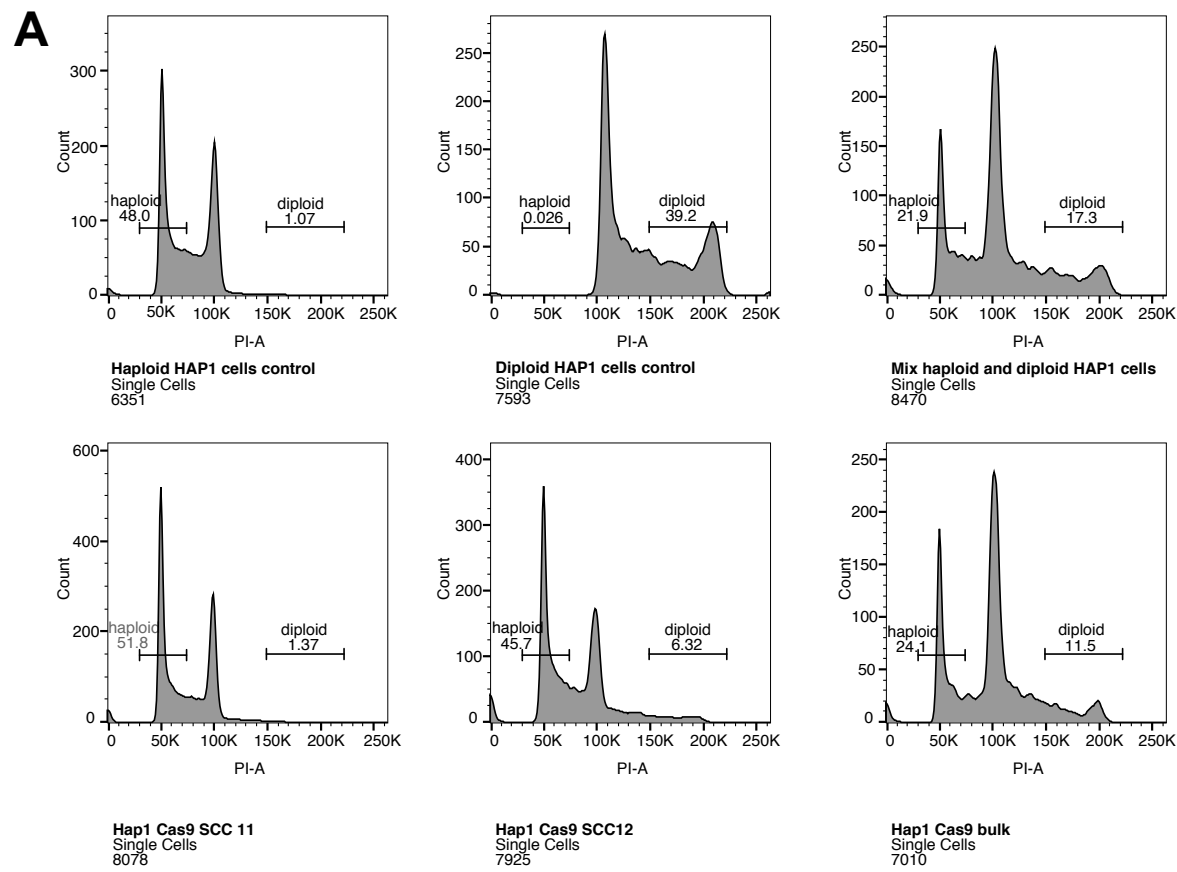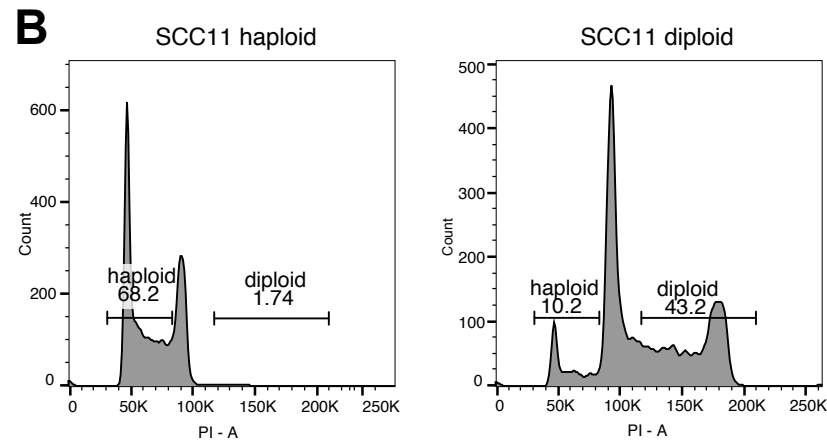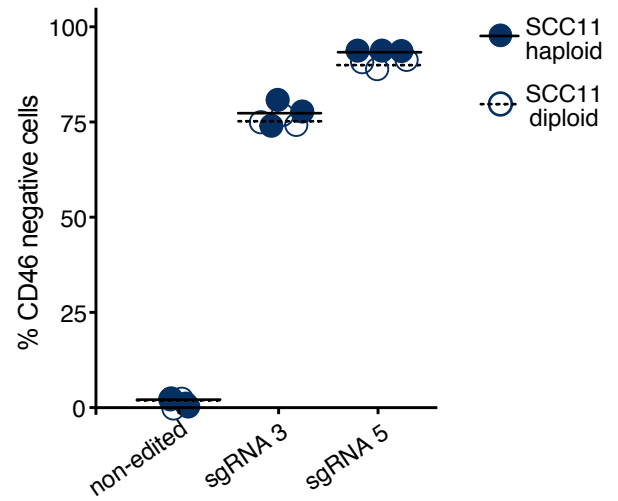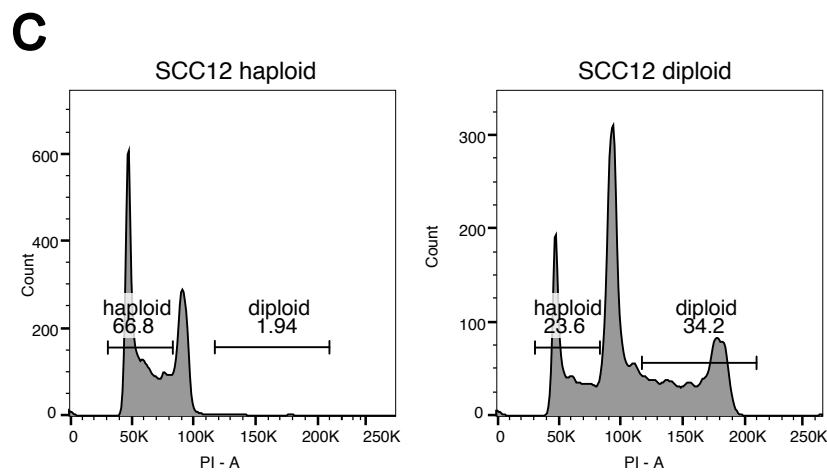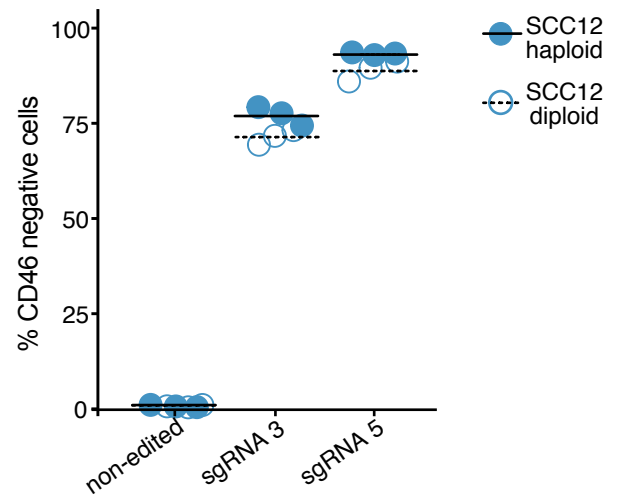

**Figure S3**

Supplement: Supplementary file 7 — Additional file 7: Figure S3. DNA content analysis to determine ploidy of various HAP1 Cas9 populations. (A) HAP1 Cas9 bulk and HAP1 Cas9 SCC11 and SCC12 cells were stained for DNA content using Nicoletti buffer and FACS analysis. The percentage of G1 haploid cells and G2 diploid cells are indicated for each cell population (n = 2 for each condition). (B-C) Enriched haploid and diploid populations of the HAP1 Cas9 SCC11 (B) and Cas9 SCC12 (C) cell lines were obtained by FACS sorting. Subsequently, haploid and diploid populations were independently transduced with the HDCRISPRv1 vector expressing sgRNAs targeting the surface marker CD46 and editing efficiency was directly compared in the haploid and diploid population of the same cell line. Non-edited samples of the respective cell lines served as a control. Lines represent the mean of three independent experiments for each condition. [file 12915_2020_905_MOESM7_ESM.pdf]

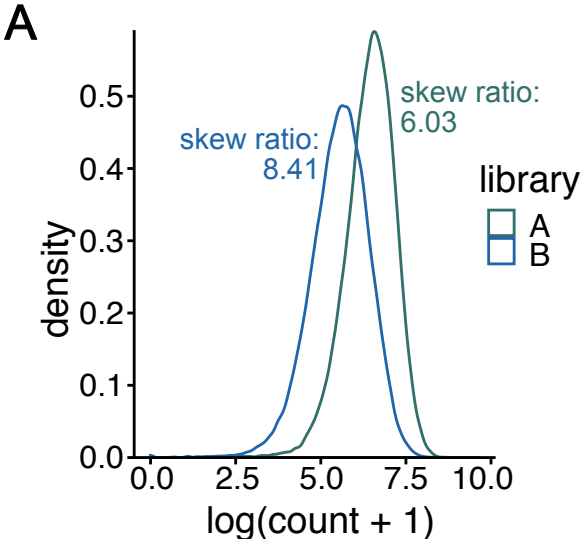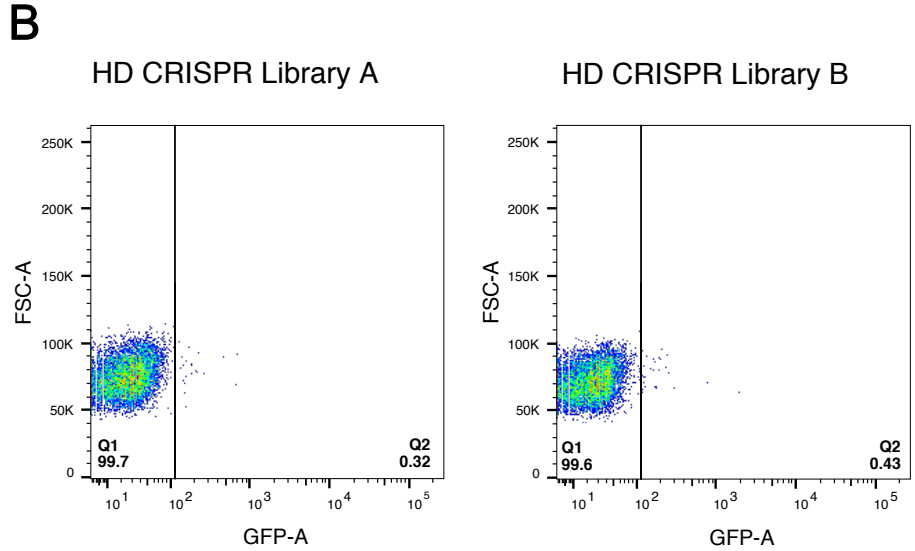

**Figure S4**

Supplement: Supplementary file 8 — Additional file 8: Figure S4. Cloning quality control of the HD CRISPR library. (A) Distribution of sgRNA read counts for the HD CRISPR plasmid library preparations. Skew ratios were determined as the quotient of the top 10 quantile divided by the bottom 10 quantile. (B) FACS analysis of GFP expression upon transfection of the HD CRISPR Library A and B plasmid pools to address the presence of remaining GFP stuffer (n = 3 for each condition). [file 12915_2020_905_MOESM8_ESM.pdf]

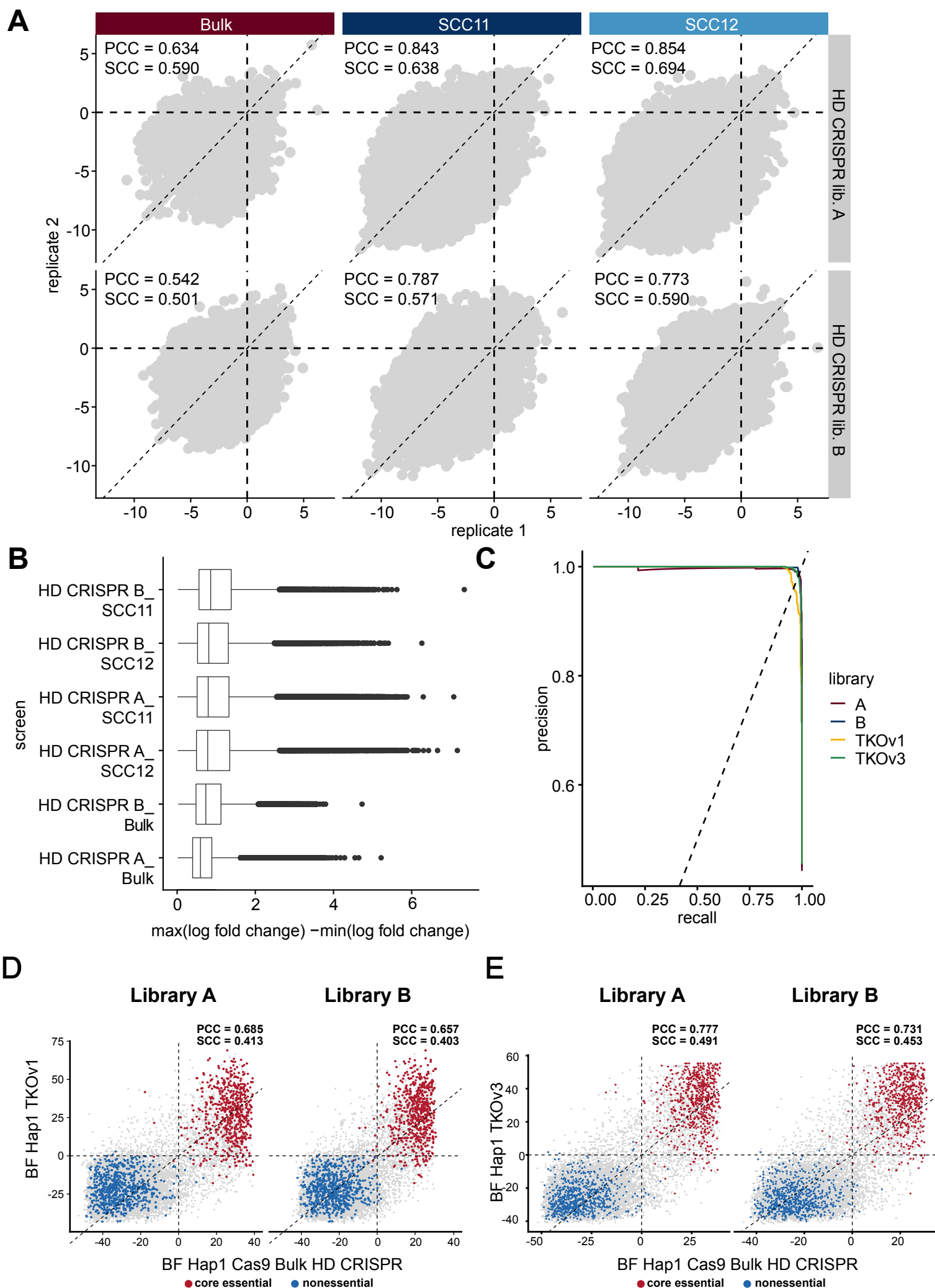

Figure S5

Supplement: Supplementary file 9 — Additional file 9: Figure S5. Reproducibility of negative selection screens with the HD CRISPR library. (A) Scatter plots showing the reproducibility of sgRNA phenotypes across biological replicates in screens with the HD CRISPR library. Each column includes screens performed in a bulk cell population (left) or in selected single cell clones with high Cas9 activity (middle and right). The top and bottom rows include screens with the HD CRISPR sub-libraries A and B, respectively. (B) Boxplot representing the distribution of the differences of the maximal and the minimal log2 fold change of guides targeting the same gene in individual screens. For each gene the difference between the maximal and the minimal sgRNA log2 fold change was calculated. This process was repeated for both HD CRISPR sublibraries using the phenotypes derived from screens in bulk population and single cell clones. Guides targeting the same gene result in similar log2 fold changes with a median difference of the maximal and the minimal log2 fold change smaller 1 for all screens. (C) Precision-recall-curve analysis for reference core essential and nonessential gene sets (Hart et al., 2015, Hart et al., 2017) of screens conducted in the HAP1 Cas9 bulk population using either the HD CRISPR Library A or B and two published CRISPR screens conducted in HAP1 cells using either the TKOv1 or TKOv3 library (Hart et al., 2017) as a reference. (D) Hit calling of the HD CRISPR Libraries A and B in comparison with a CRISPR screen conducted in HAP1 cells by Hart et al. (2017) using the TKOv1 library. (E) Hit calling of the HD CRISPR Libraries A and B in comparison with a CRISPR screen conducted in HAP1 cells by Hart et al. (2017) using the TKOv3 library. PCC = Pearson Correlation Coefficient, SCC = Spearman Correlation Coefficient. [file 12915_2020_905_MOESM9_ESM.pdf]

A

Hit calling with BAGEL

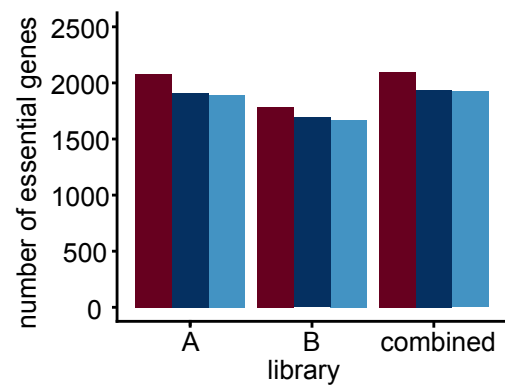

B

Hit calling with MAGeCK RRA

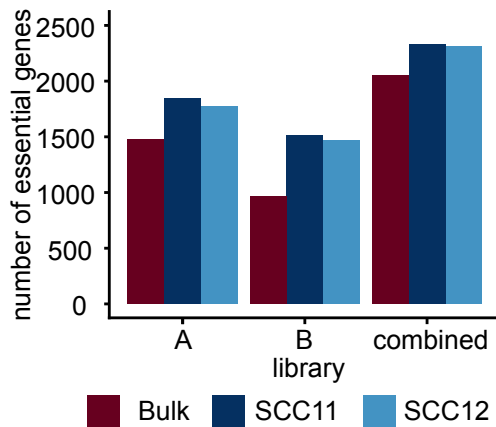

C

Hit calling with gscorend

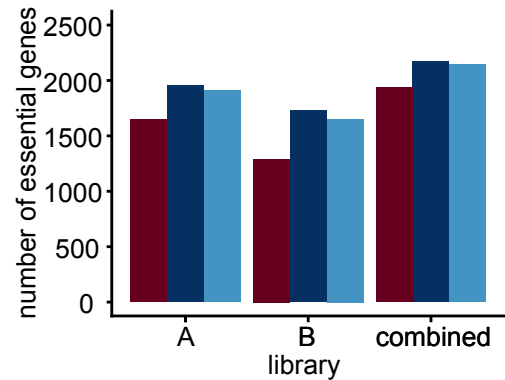

D

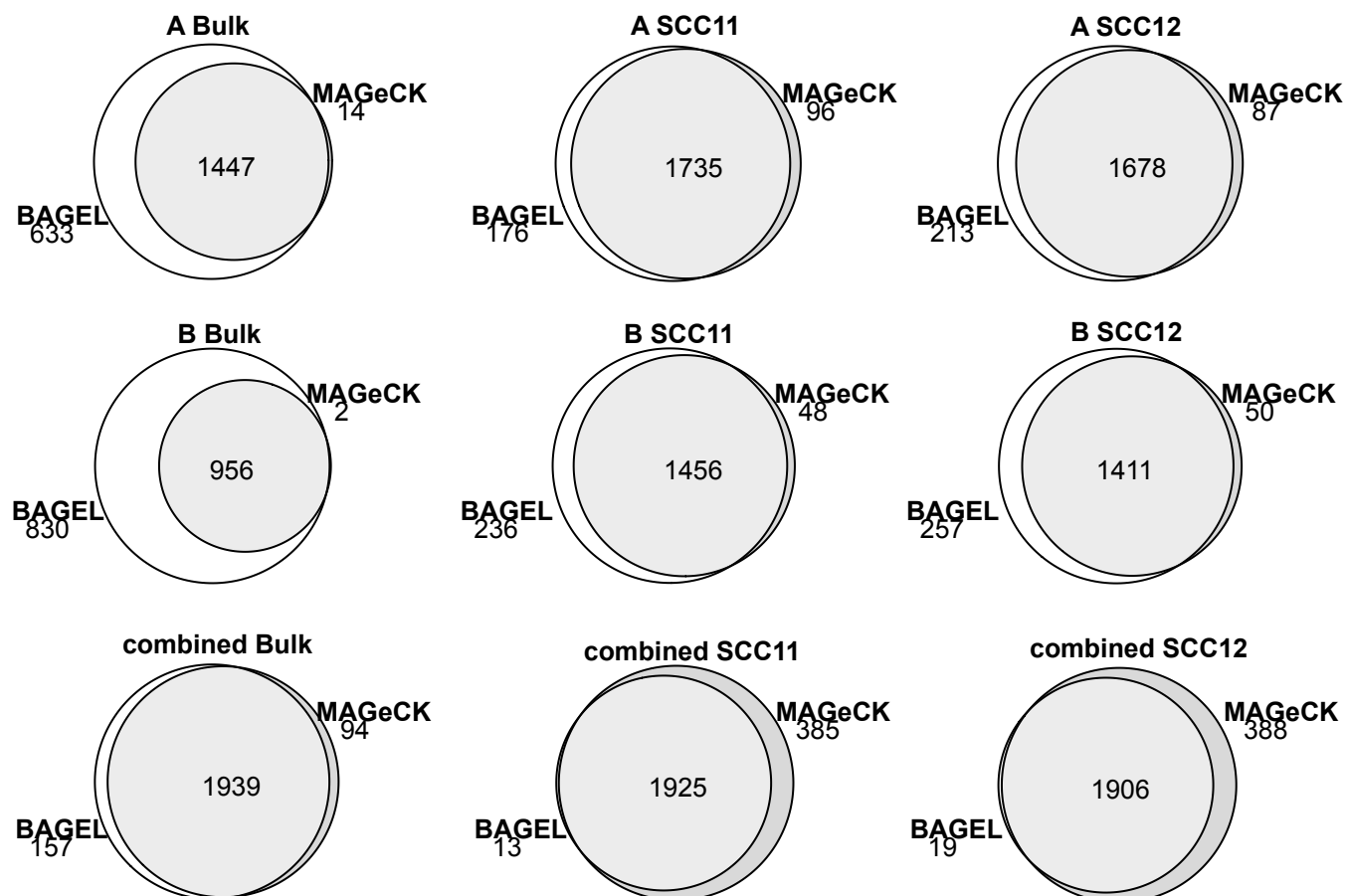

Figure S6

Supplement: Supplementary file 11 — Additional file 11: Figure S6. Hit detection in screens with the HD CRISPR library. (A) Number of hits determined using BAGEL [32] at a strict Bayes factor cutoff (BF > 6) in different screens conducted with the HD CRISPR library. (B) Number of essential genes determined using MAGeCK RRA [42] at 5% FDR in different screens conducted with the HD CRISPR library. (C) Number of essential genes determined using gscreend [43] at 5% FDR in different screens conducted with the HD CRISPR library. (D) Venn diagrams showing the overlap between essential genes determined using either BAGEL or MAGeCK RRA for each screen. [file 12915_2020_905_MOESM11_ESM.pdf]

**A**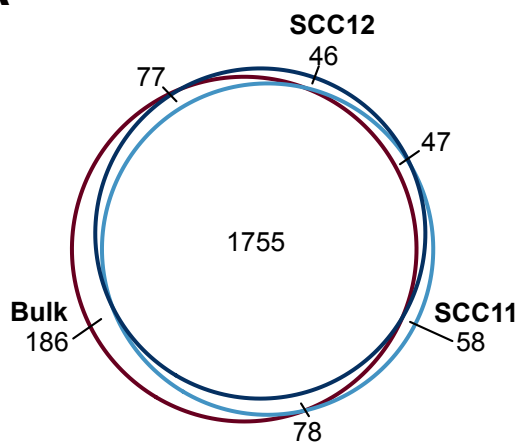**B**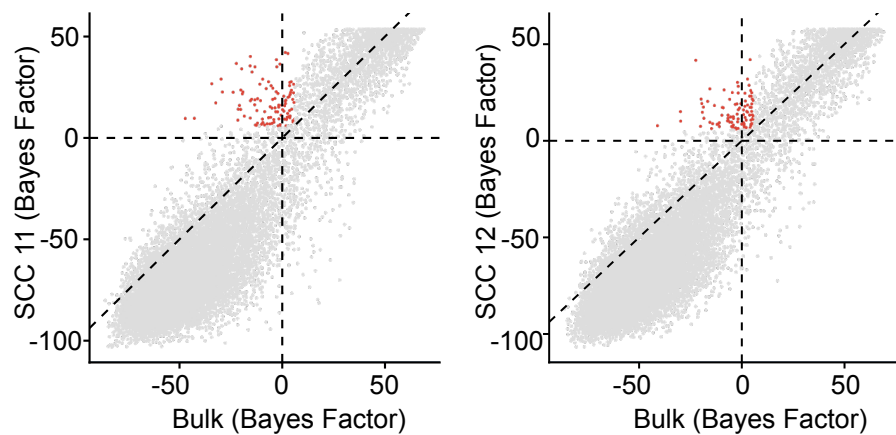**C****sub-library A**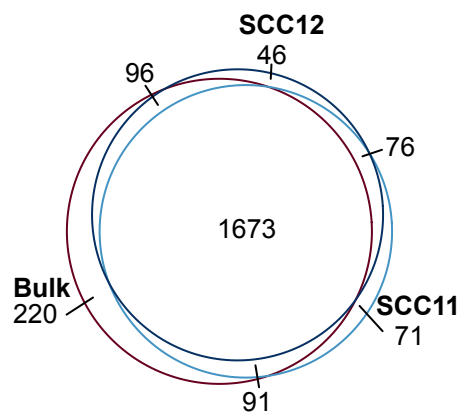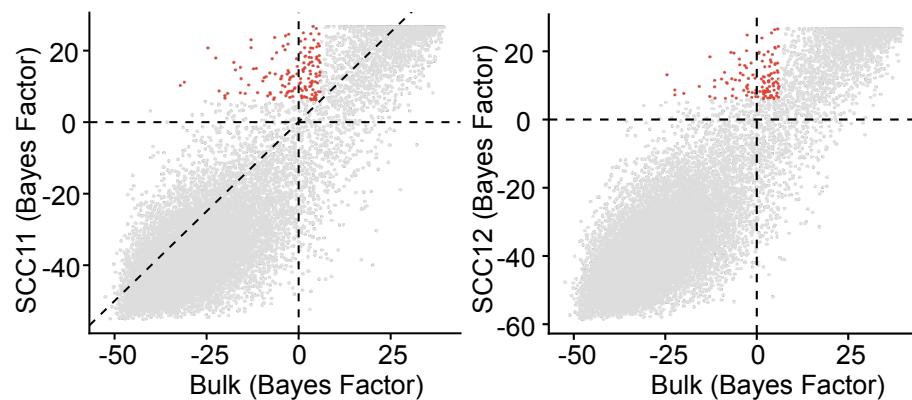**D****sub-library B**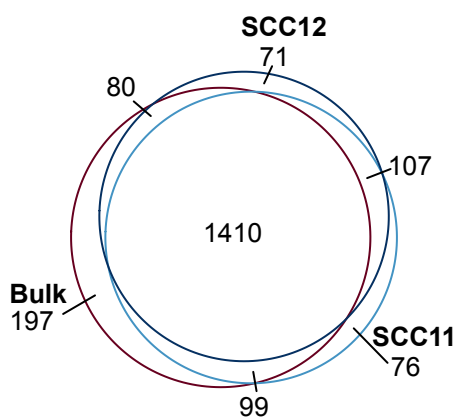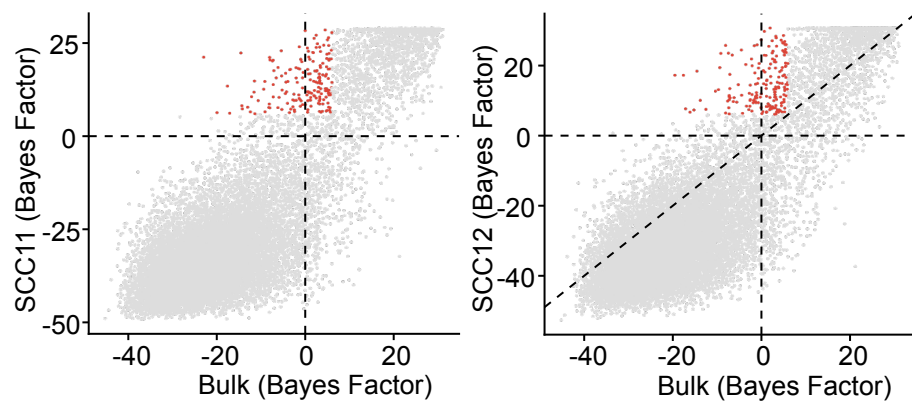**Figure S7**

Supplement: Supplementary file 12 — Additional file 12: Figure S7. Essential genes are highly consistent between HAP1 Cas9 bulk population and single cell clones. A) Venn diagram showing essential gene overlap between a HAP1 bulk Cas9 population and two single cell clones that were selected for high Cas9 activity. Gene essentiality was determined using BAGEL with a Bayes Factor cutoff of 6 (see [17]). The combined HD CRISPR library with 8 sgRNAs per gene was used for essential gene inference. (B) Quantitative comparison of BAGEL Bayes Factors for each gene between the HAP1 bulk Cas9 population and selected single cell clones SCC11 and SCC12. Each dot represents a gene in the HD CRISPR library. Red dots indicate essential genes that are private to a single cell clone. The dashed diagonal is the identity line. (C) Venn diagram (left) and scatter plots (middle and right) showing essential gene overlap between a HAP1 bulk Cas9 population and two single cell clones in screens using the HD CRISPR sub-library A. (D) Venn diagram (left) and scatter plots (middle and right) showing essential gene overlap between a HAP1 bulk Cas9 population and two single cell clones in screens using the HD CRISPR sub-library A. [file 12915_2020_905_MOESM12_ESM.pdf]

**A**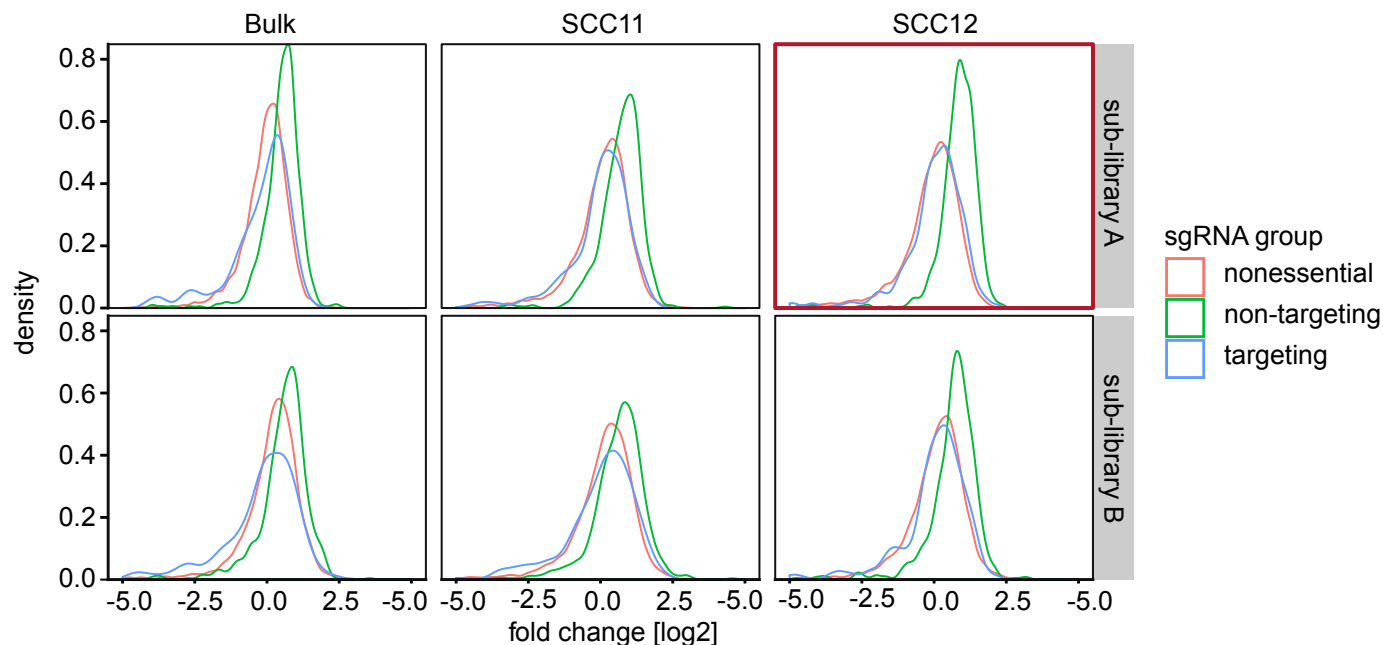**B**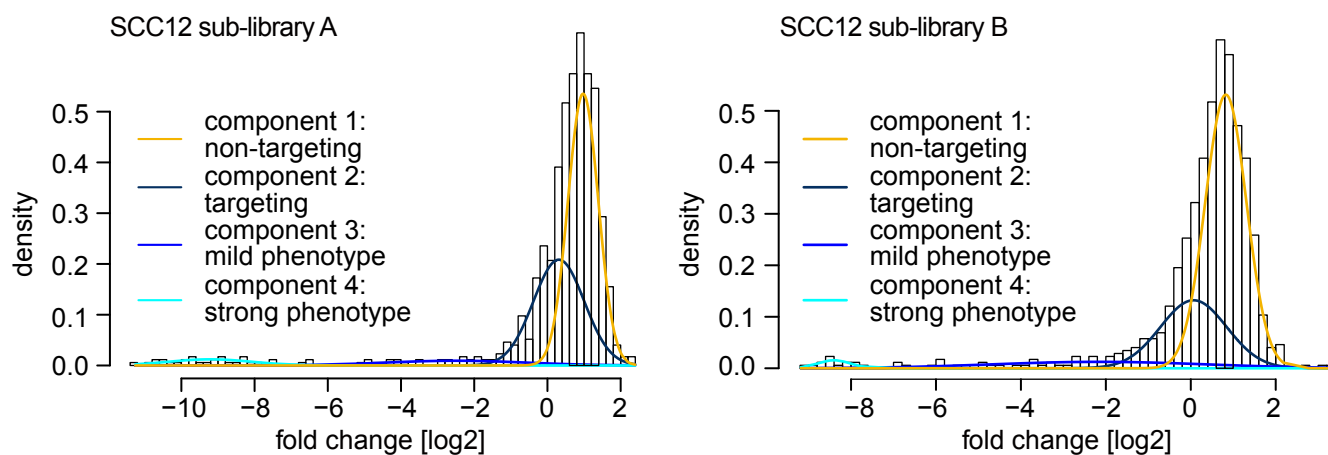**C**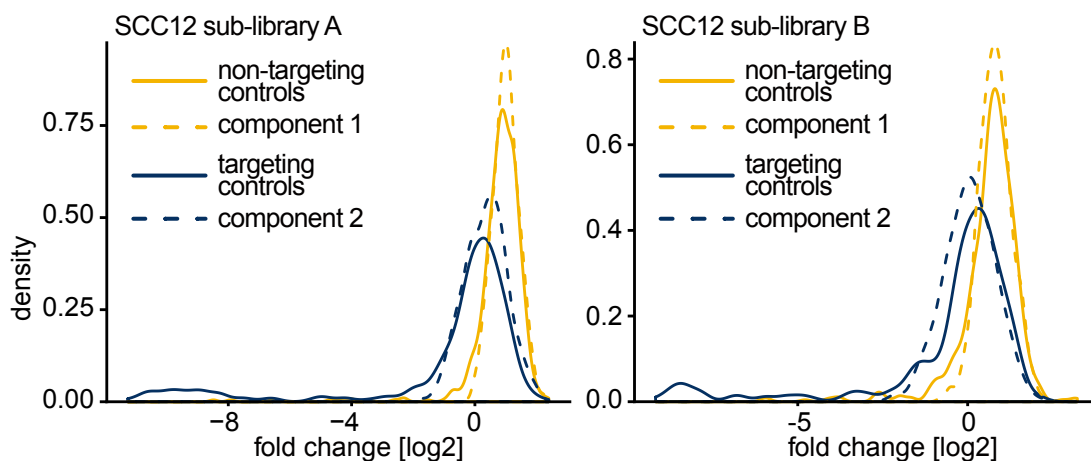**D**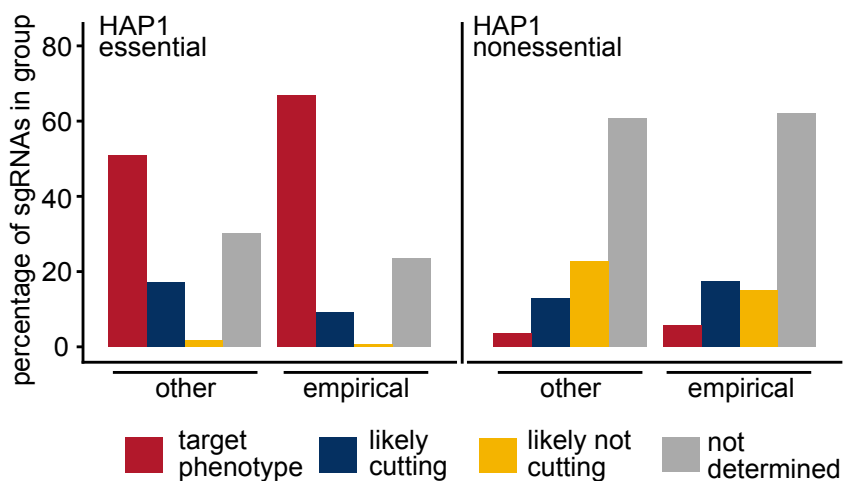**Figure S8**

Supplement: Supplementary file 13 — Additional file 13: Figure S8. Prediction of sgRNA DNA cutting activity based on control phenotypes. (A) Log2 fold change phenotype distributions for sgRNAs targeting nonessential genes (red) as well as targeting (blue) and non-targeting control sgRNAs (green) across different screens conducted with the HD CRISPR library. The screen with the HD CRISPR library A in HAP1 single cell clone SCC12, which was used for subsequent analyses, is highlighted in red. (B) Fit of a Gaussian mixture model with 4 components for screens in SCC12. Components 1 (yellow) and 2 (blue) represent non-targeting and targeting sgRNAs, respectively. Components 3 and 4 capture the phenotypes of sgRNAs with moderate and severe viability phenotypes. (C) Comparison of true fold change distributions of targeting and non-targeting sgRNAs (solid line) to the distributions estimated by the mixture model components (dashed lines) for both HD CRISPR libraries A and B. (D) Number of sgRNAs associated with each phenotype group targeting essential genes according to MAGeCK analysis. For this representation components 3 and 4 are combined in the red group ‘target phenotype’. sgRNAs are stratified based on their design: ‘empirical essential’ sgRNAs target context-specific essential genes and were selected for the HD CRISPR library based on their previous on-target phenotypes. ‘Empirical nonessential’ sgRNAs are part of previously published libraries and target broadly nonessential genes. They were selected based on their lack of outlier phenotypes. De novo sgRNAs were designed using the software cld [34]. (E) Similar plot as D showing sgRNAs of the HD CRISPR library B associated with each phenotype group. [file 12915_2020_905_MOESM13_ESM.pdf]

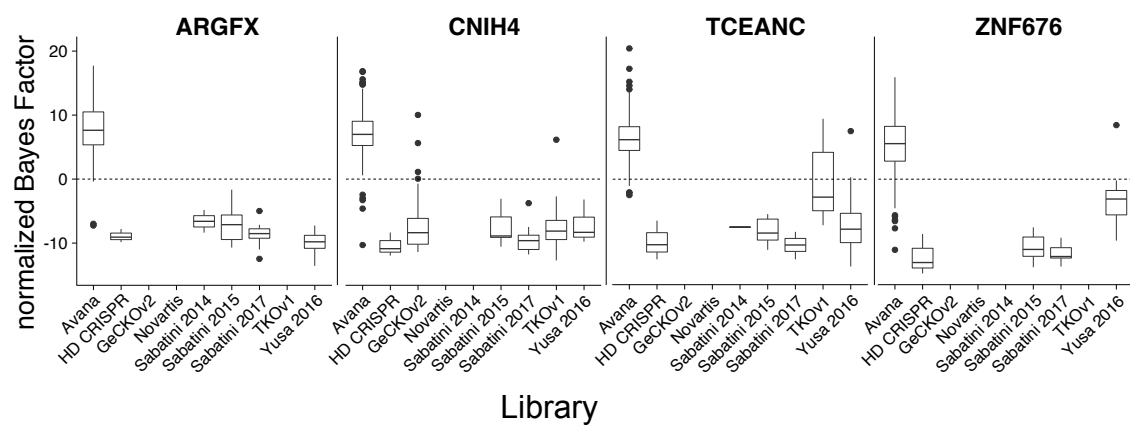

**Figure S9**

Supplement: Supplementary file 14 — Additional file 14: Figure S9. HD CRISPR Library design strategy does not enrich for sgRNAs with strong phenotypes presumably caused by off-target effects. Bayes Factor analysis of selected HAP1 context-dependent nonessential genes across different screens conducted with various genome-scale CRISPR libraries in cancer cell lines. [file 12915_2020_905_MOESM14_ESM.pdf]

**A**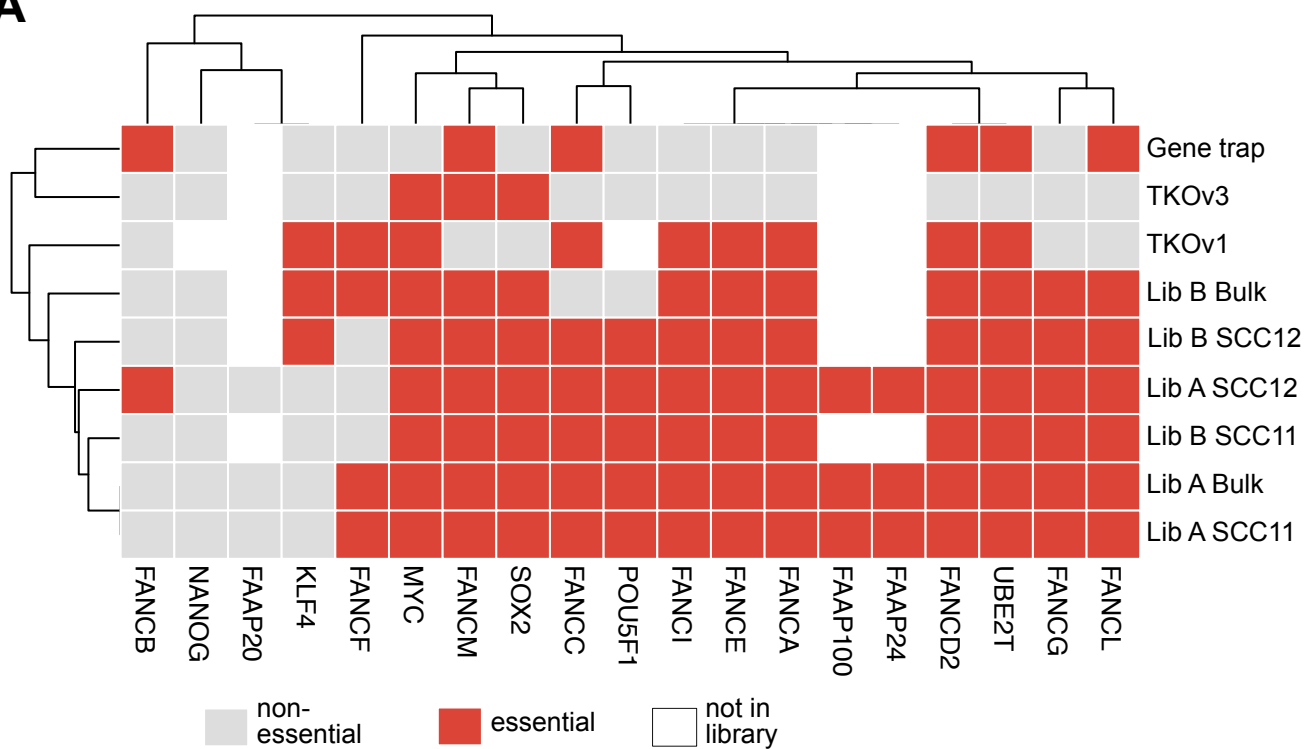**B**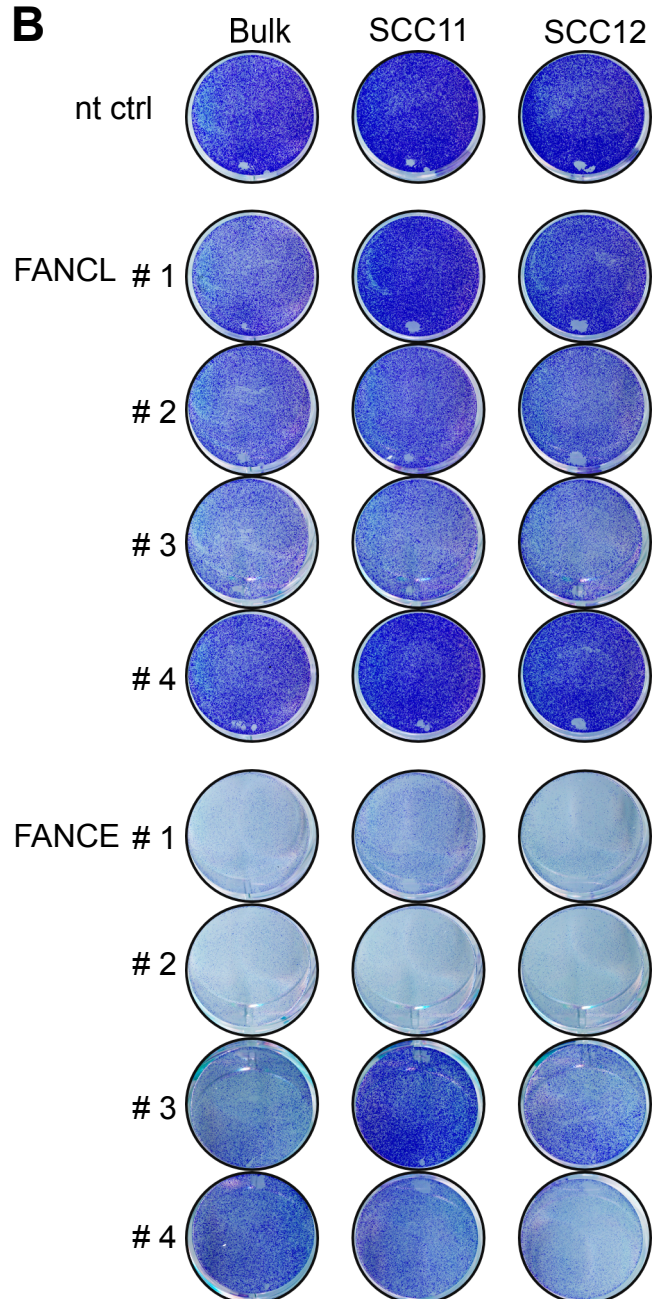**C**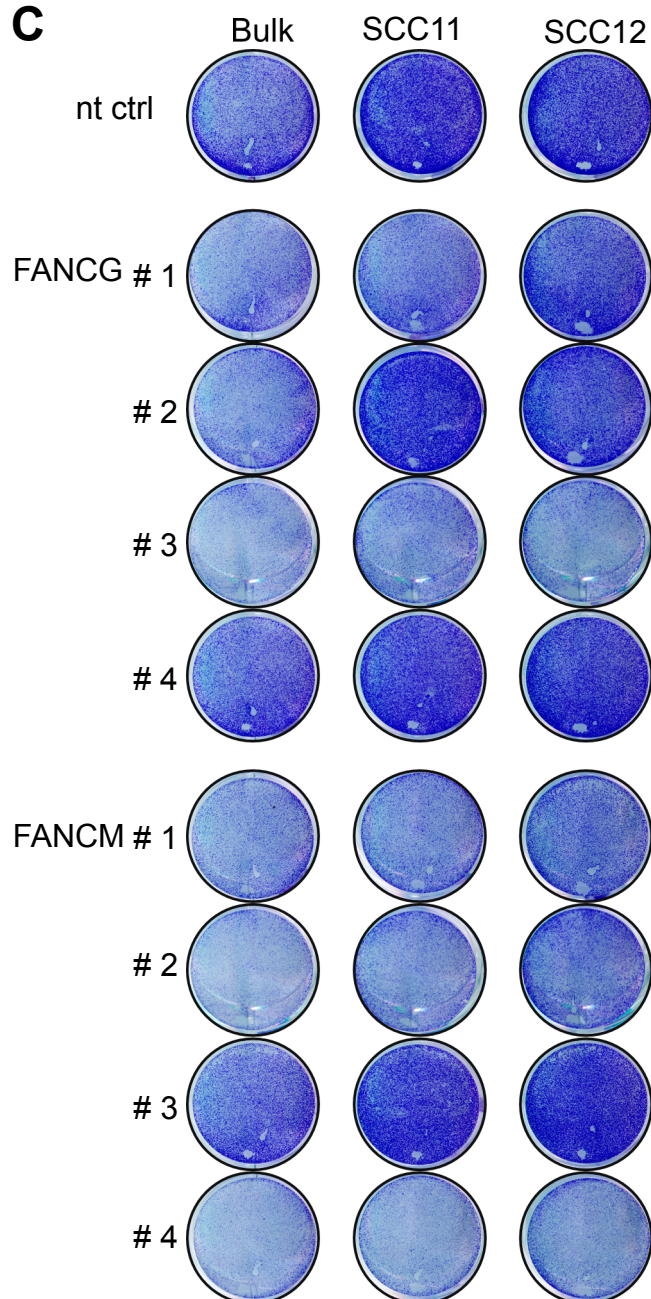**Figure S10**

Supplement: Supplementary file 15 — Additional file 15: Figure S10. The identified dependency of HAP1 cells on pluripotency genes and the Fanconi anemia pathway is only partially detected in other published screens conducted in HAP1 cells. (A) Essentiality of Yamanaka factors and Fanconi anemia pathway members in individual HD CRISPR HAP1 and previously published TKO HAP1 CRISPR screens. Red boxes indicate that the gene was found essential and gray indicates non-essentiality. White boxes represent genes that are not targeted by the respective library. (B) siRNA knockdown of the FANCL and FANCE expression using four individual siRNAs each in the HAP1 Cas9 bulk and HAP1 Cas9 SCC11 and SCC12 cell lines. A pool of non-targeting siRNAs was used as a control. Surviving cells were stained with crystal violet solution. (C) Same as (B) for FANCG and FANCM expression. Representative images from four independent experiments conducted for each condition are shown. nt ctrl = non targeting control. [file 12915_2020_905_MOESM15_ESM.pdf]
